# Supplementary figures and images for: Hierarchical cortical transcriptome disorganization in autism
Source: Mol Autism. 2017 Jun 21;8:29. doi: 10.1186/s13229-017-0147-7 (PMC5480153; doi:10.1186/s13229-017-0147-7)

# Scale Free Topology Model Fit

Scale Free Topology Model Fit

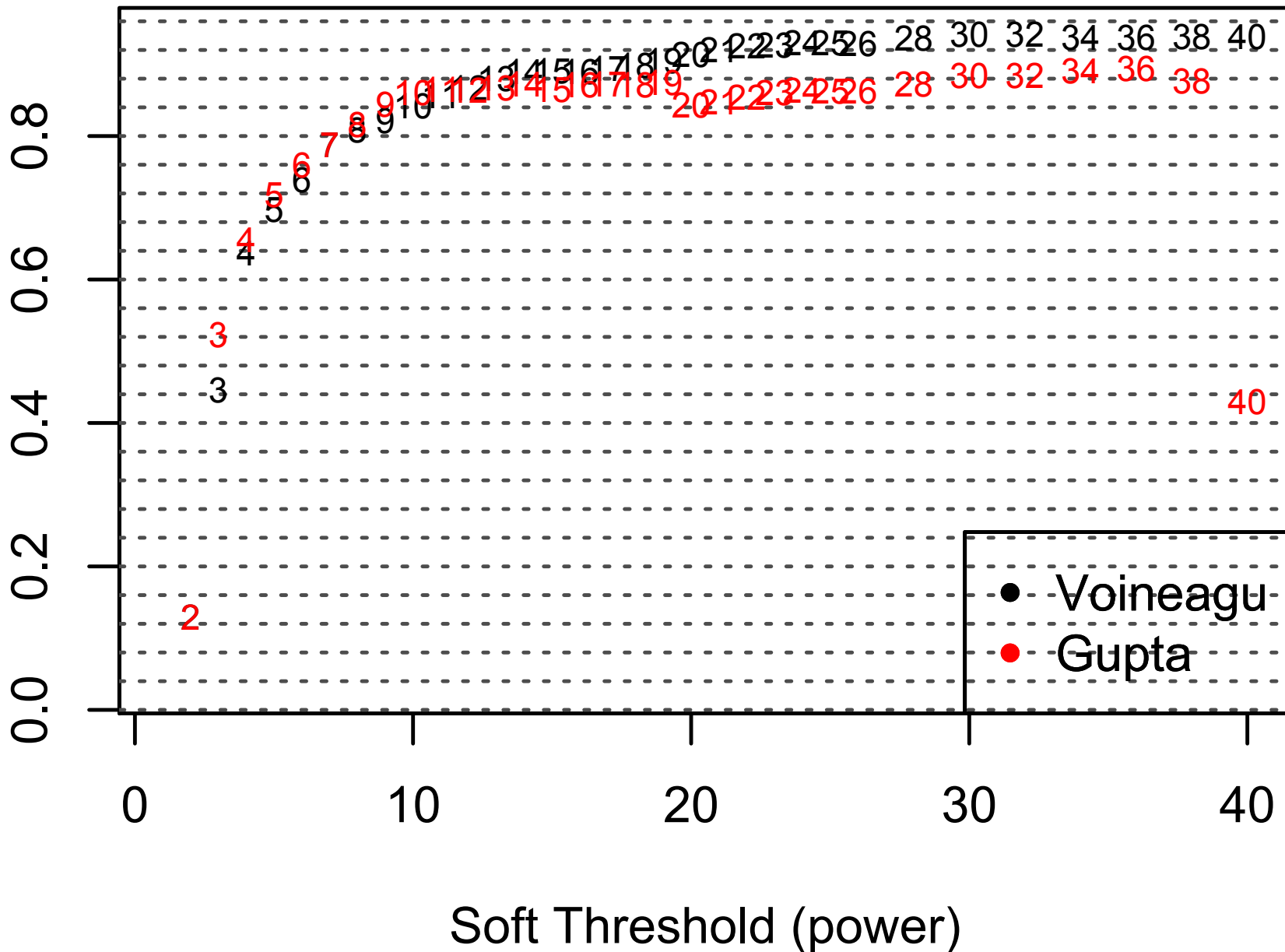

Supplement: Supplementary file 1 — Scale-free topology model fit across a range of soft power thresholds. This plot shows the scale-free topology model fit scores (R 2) across a range of soft power thresholds. This analysis is done in order to choose a soft-power threshold to use in the main analyses. As a rule, we picked the soft power threshold whereby scale-free topology model fit R 2 is maximum and begins to plateau (i.e., soft power = 14). (PDF 1946 kb) [file 13229_2017_147_MOESM1_ESM.pdf]

# Voineagu

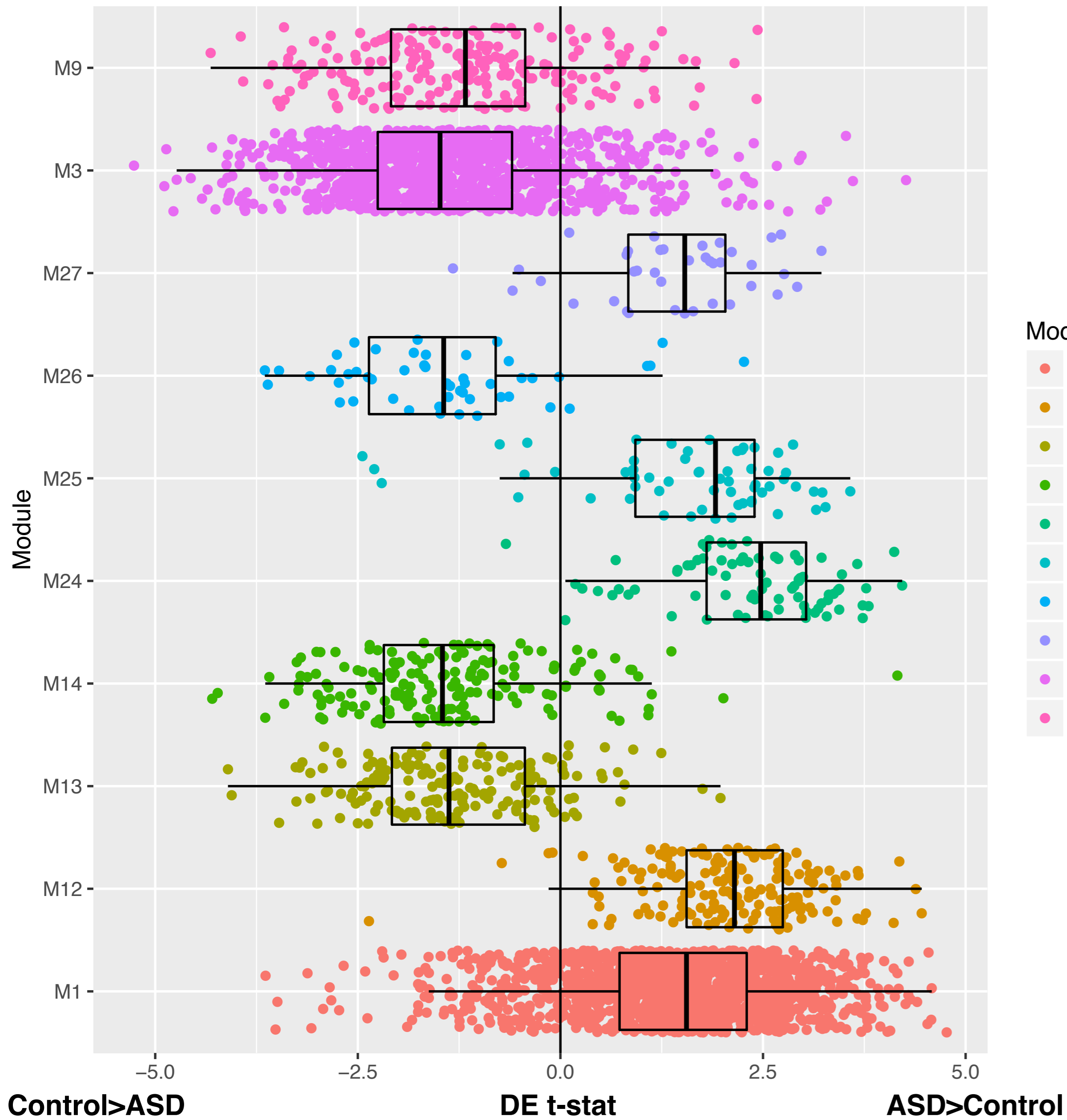

# Gupta

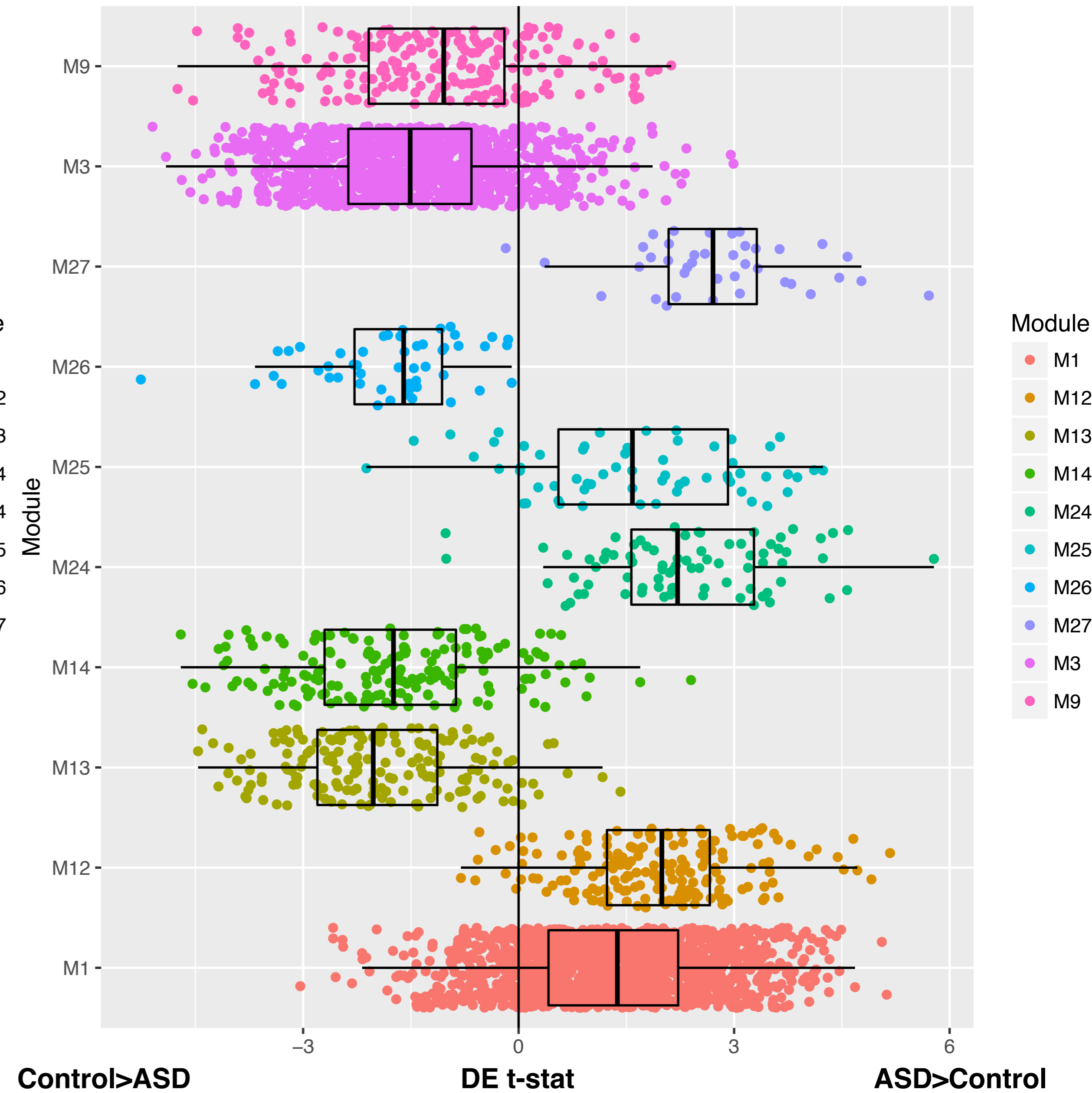

Supplement: Supplementary file 6 — Differential expression load within replicably dysregulated co-expression modules. This plot shows strength of differential expression (DE) for each gene within the 10 replicably dysregulated co-expression modules. DE strength is quantified continuously as the effect size (t stat) from the DE gene-level analyses. All modules show a substantial shift in DE signal in the direction congruent with the label of “upregulated” (ASD > Control) or “downregulated” (Control > ASD) given to each module. (PDF 107 kb) [file 13229_2017_147_MOESM6_ESM.pdf]

**A****Voineagu Control**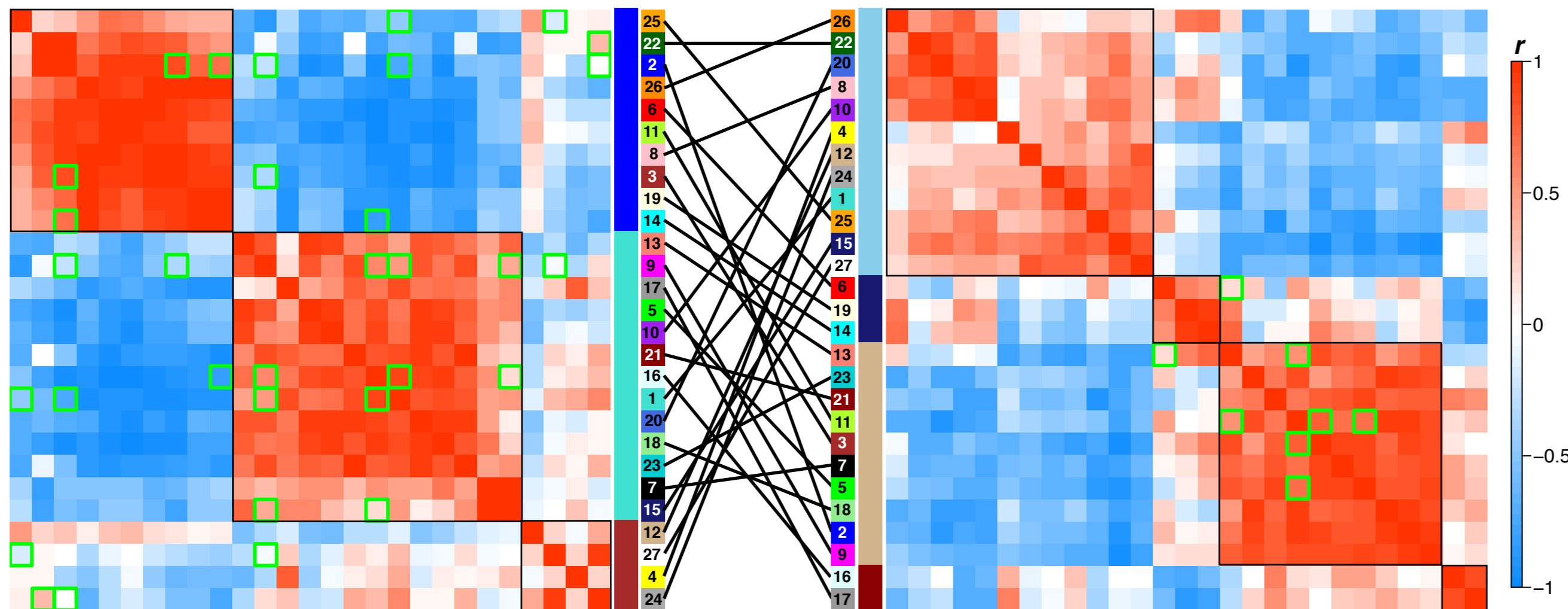**Gupta Control**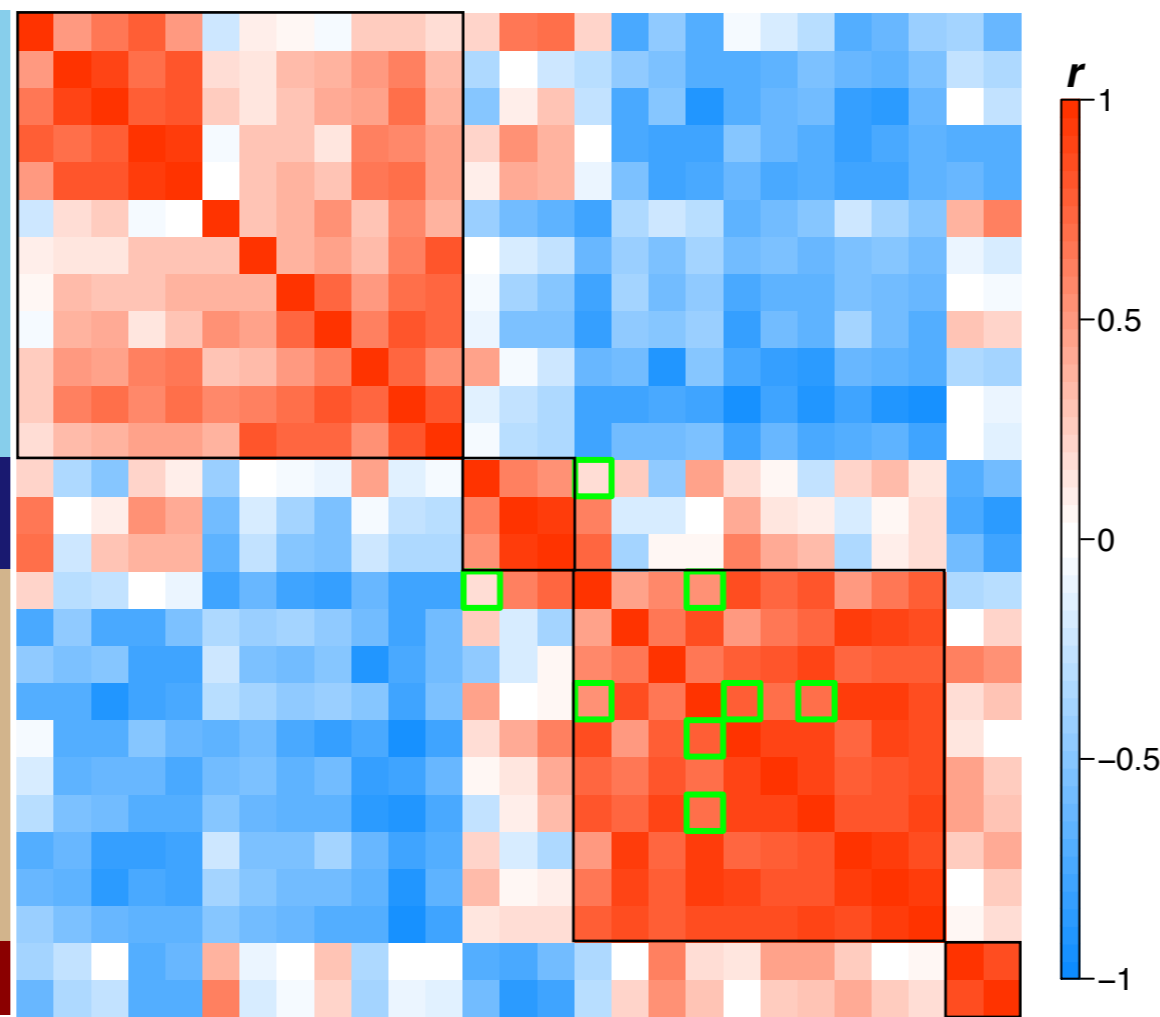**C****Average Preservation ( $D = 0.69$ )**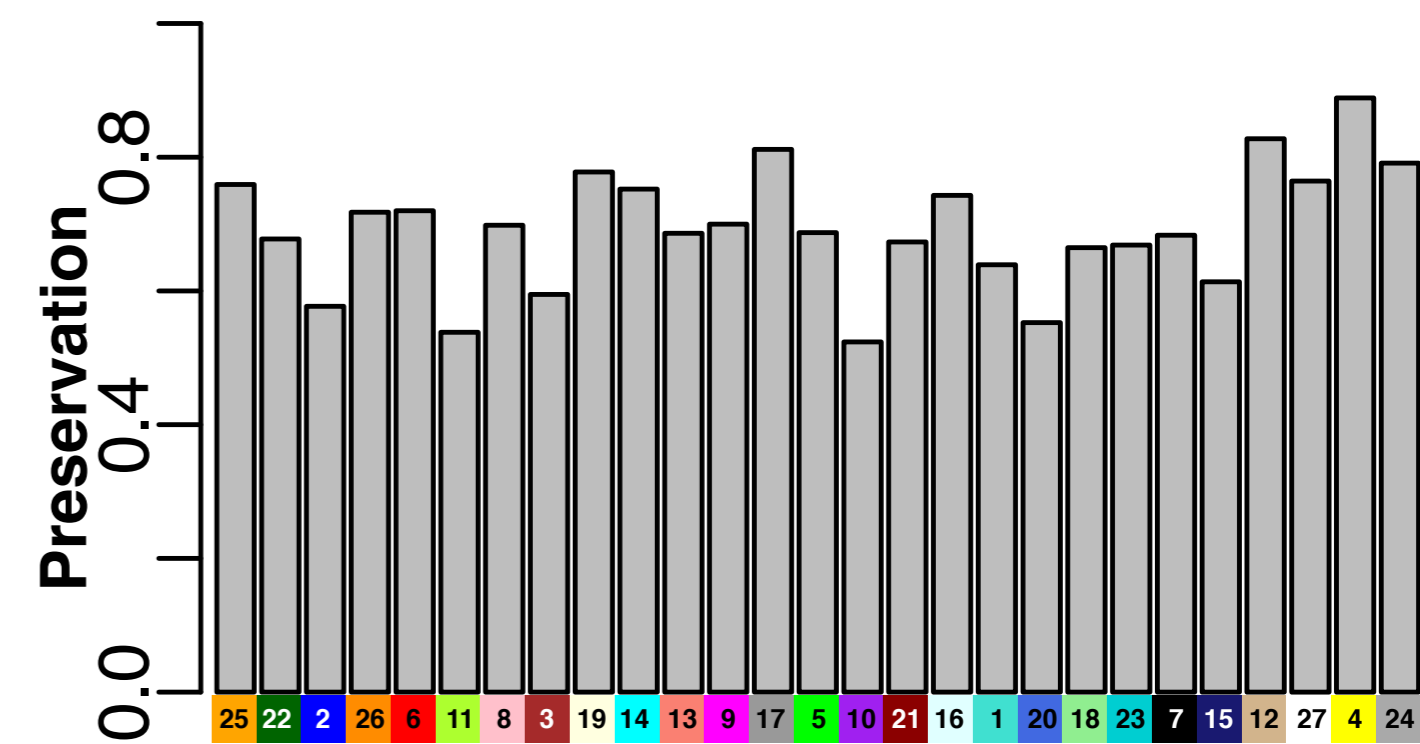**B**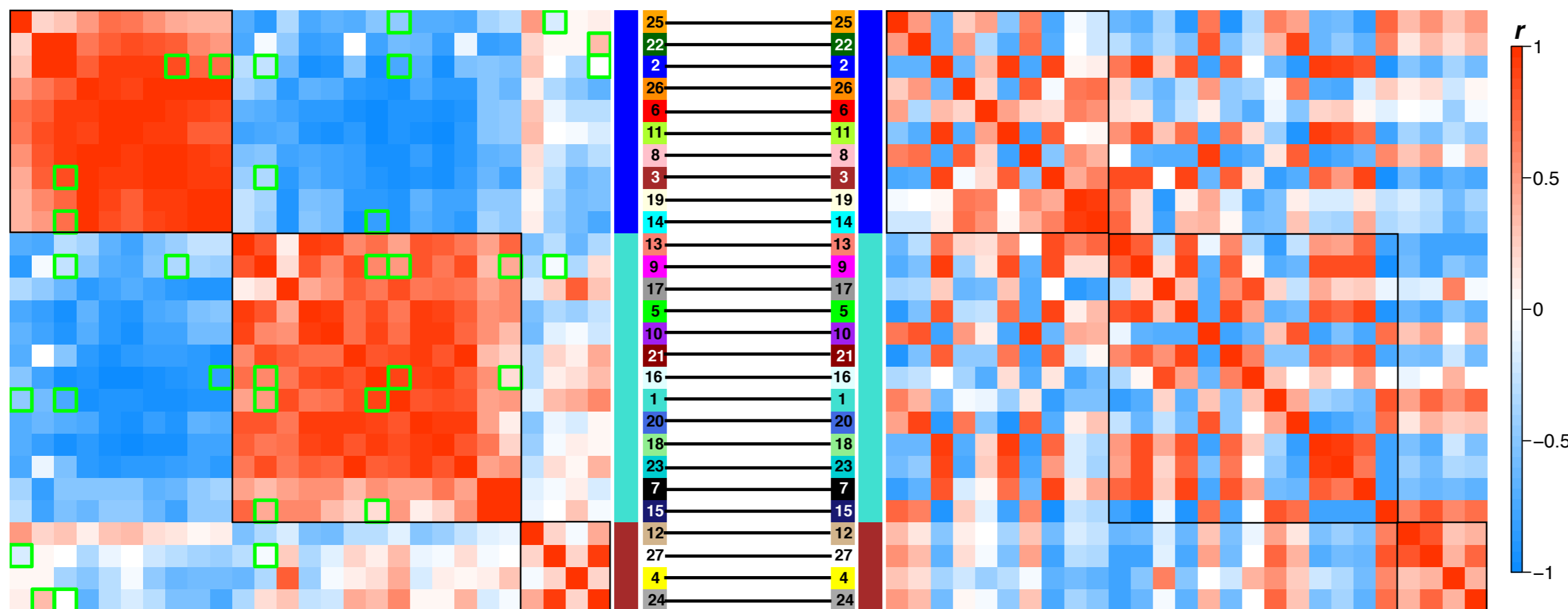**D****Preservation**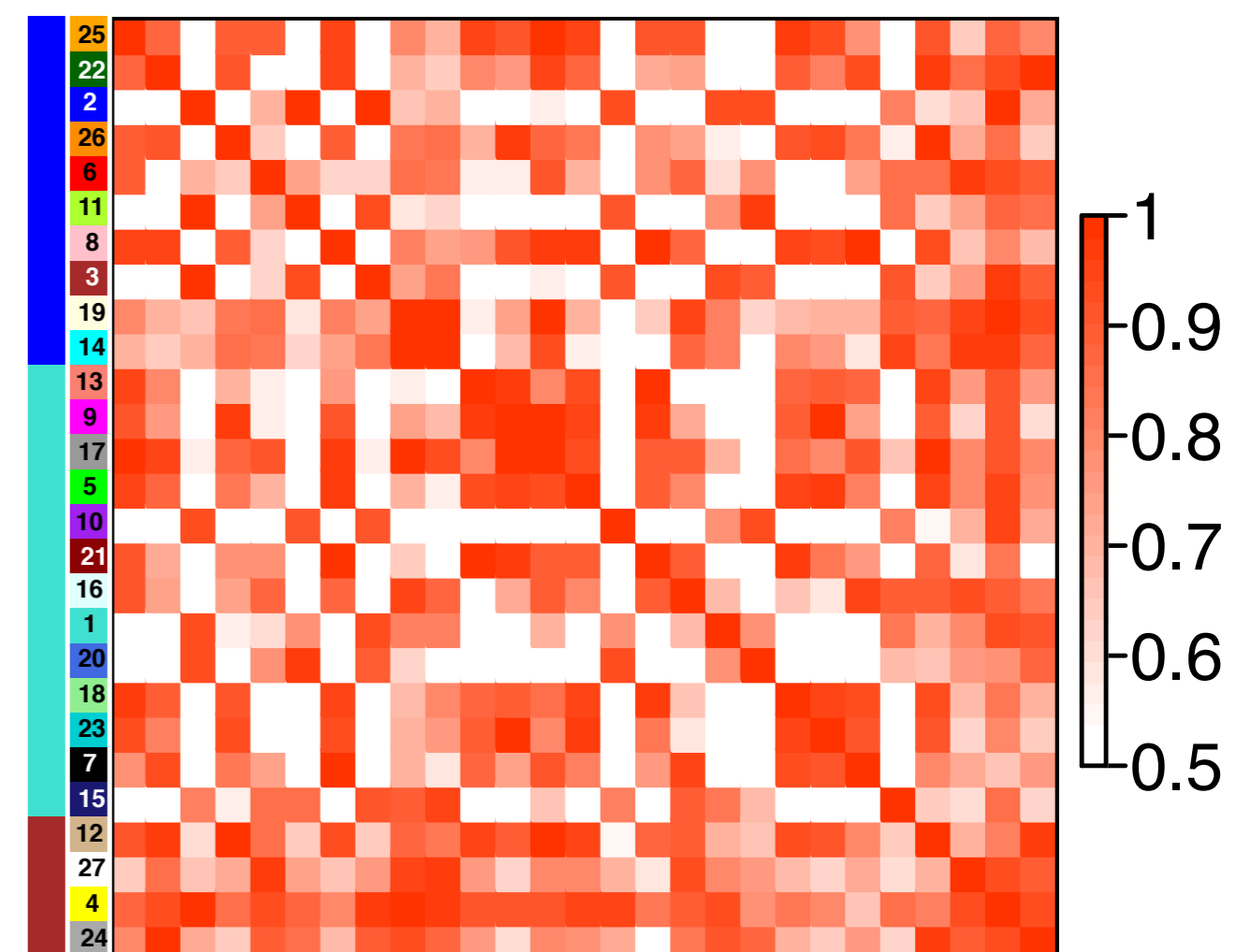

Supplement: Supplementary file 7 — Preservation of eigengene networks in the TD group. Panel A shows the eigengene networks for Voineagu and Gupta datasets when the rows and columns of the matrix are ordered by meta-module clustering. Panel B shows the matrices when ordered only by the Voineagu TD dataset clustering. Panel C shows average preservation levels across each module. Panel D shows preservation for all pairwise module comparisons. The plots in panels C and D were made using a modified version of the plotEigengeneNetworks function in the WGCNA R library. We modified this function to use ME robust partial correlation matrices. (PDF 331 kb) [file 13229_2017_147_MOESM7_ESM.pdf]
